# Neuropsychological outcomes following awake surgery in adult glioma patients: A systematic review

**DOI:** 10.1093/nop/npaf075

**Published:** 2025-07-30

**Authors:** Laura Amores-Carrera, Isabel Martín-Monzón

**Affiliations:** Grupo de Investigación Neurociencia del Bienestar, Departamento de Psicología Experimental, Facultad de Psicología, Universidad de Sevilla, Sevilla (España), Spain; Grupo de Investigación Neurociencia del Bienestar, Departamento de Psicología Experimental, Facultad de Psicología, Universidad de Sevilla, Sevilla (España), Spain

**Keywords:** awake craniotomy, cognitive functions, direct electrical stimulation, neuroplasticity, postoperative neuropsychological outcomes

## Abstract

**Background:**

Awake craniotomy is essential for glioma resection in functionally integrated brain regions, allowing real-time monitoring to reduce cognitive and emotional deficits. Although widely used, its long-term neuropsychological effects remain debated. This systematic review aims to investigate cognitive and emotional outcomes after awake brain surgery and the factors that influence recovery, including extent of resection, follow-up timing, and neural plasticity.

**Methods:**

This systematic review analyzes 34 studies on pre- and postoperative functional outcomes in glioma patients undergoing awake surgery. Following PRISMA guidelines, studies were selected with adult glioma patients (WHO grade I-IV) who had neuropsychological assessments before and after surgery. Data on preoperative cognitive profiles, recovery trajectories, and follow-up durations were examined, focusing on methodological consistency and assessment tools.

**Results:**

The findings demonstrated substantial variability in functional outcomes, with many patients recovering within 3 to 6 months post-surgery, while others experienced persistent deficits. Functional recovery was influenced not only by the extent of tumor resection but also by network-level reorganization. Methodological inconsistencies in neuropsychological assessments highlighted the need for standardized, personalized evaluation protocols, emphasizing the importance of comprehensive functional assessments.

**Conclusions:**

This review emphasizes the shift from a localized cortical approach to a dynamic, network-based view of cognitive and emotional recovery. It calls for standardized, personalized neuropsychological assessments to optimize rehabilitation, along with extended follow-ups, and multidisciplinary care for long-term quality of life. Future research should refine assessment methods and strategies to better understand neuroplasticity and improve clinical outcomes in neuro-oncology.

**Key Points**

- This review analyzes neurocognitive and emotional outcomes after awake glioma surgery.
- Cognitive recovery and quality of life improve with tailored pre/post assessments.
- Functional network reorganization depends on the extent of resection and neuropsychological assessment.

**Importance of the Study**

This systematic review provides a comprehensive analysis of neuropsychological outcomes following awake craniotomy for glioma resection, emphasizing the need for extended cognitive and emotional monitoring. Findings from 34 studies show that most patients experience minimal or non-cognitive or emotional decline, with many recovering within 3 to 6 months. Some patients even exhibit improvements in executive control, as well as in sensory-motor functions, memory, reading, emotion recognition, and theory of mind. The review emphasizes the need for standardized yet patient-specific neuropsychological assessments to optimize recovery. Additionally, the extent of resection (EOR) was maximized while preserving critical neural structures, leading to favorable oncological outcomes. Reported survival rates exceeded 90%, reinforcing the benefits of awake mapping techniques. This work highlights the critical balance between tumor resection and cognitive-emotional preservation, advocating for long-term follow-up to enhance patient care and quality of life.

Awake craniotomy has emerged as a transformative surgical approach for glioma resection in brain regions involved in cognitive and neurological functions, where preservation of these functions is crucial. This technique enables intraoperative monitoring of critical brain networks through real-time patient feedback, allowing neurosurgeons to navigate complex cortical and subcortical areas while minimizing the risk of permanent deficits.^1^ Integrating the principles of neuroplasticity, and meta-networking theory, awake craniotomy offers an advanced approach to preserving both neural function and the overall quality of life for patients.^1–3^

Neuroplasticity, the brain’s remarkable ability to reorganize and adapt to damage, underpins the success of awake craniotomy. Studies have demonstrated that functional reorganization can occur both at a local and network-wide level, enabling preserved networks to compensate for resected or damaged areas.^3–5^ The advent of connectomics, the comprehensive mapping of neural networks, has further revolutionized neurosurgery by providing detailed insights into the interplay between large-scale neural networks, such as the default mode network (DMN) and the frontoparietal control network, are integral to brain function.^6,7^ These advances have allowed surgeons to tailor interventions to each patient’s unique connectome, maximizing functional preservation and recovery potential.^8^

While awake craniotomy has demonstrated significant benefits in tumor resection and functional outcomes, understanding long-term neuropsychological effects requires a systematic exploration of follow-up timing and methodologies. Studies have shown that cognitive and emotional recovery can vary widely, with critical improvements often observed within the first 3 to 6 months post-surgery but continuing for up to a year or more as neuroplastic adaptations evolve.^9^ However, inconsistencies in the timing of neuropsychological assessments, ranging from immediate postoperative evaluations to long-term follow-ups, have hindered comparisons across studies.^9,10^ These differences underscore the need for standardized follow-up protocols to assess not only early recovery but also the sustained effects of surgical interventions on quality of life.^11^

The main objective of this systematic review is to critically analyze and synthesize the existing literature on long-term neuropsychological outcomes following awake glioma surgery, with a particular focus on how differences in assessment methods, timing, and reporting contribute to variability in reported results. By doing so, we aim to highlight the current gaps and inconsistencies in the field, and to emphasize the need for standardized, longitudinal neuropsychological follow-up protocols to better understand and optimize patients’ cognitive recovery and quality of life. Our findings not only reinforce the effectiveness of awake surgery but also advocate for a shift toward long-term neuropsychological monitoring to optimize patient care and functional outcomes.

## Materials and Methods

Details of the protocol for this systematic review were registered in the PROSPERO database CRD42025641857.

### Literature Search Strategy

A systematic literature review was performed in accordance with the PRISMA guidelines (Preferred Reporting Items for Systematic Reviews and Meta-Analyses) to ensure the review methodology met quality standards.^12,13^ The databases utilized for article searches were PubMed, Scopus, Web of Science, and Embase. These databases were selected due to their comprehensive coverage of neuroscience literature.

The searching procedure was conducted systematically across the selected databases using a combination of keywords and Boolean operators: (“awake brain surgery” OR “awake craniotomy” OR “awake surgery” OR “awake brain mapping” OR “intraoperative mapping” OR “intraoperative monitoring”) AND (“neuropsychological outcomes” OR “cognitive outcomes” OR “neurocognitive outcomes” OR “emotional outcomes” OR “long-term outcome”) in different combinations. The search strategy was tailored for each database to optimize the relevance of the results obtained. The search in the databases was restricted to articles published in academic journals from 2005 to 2025, reflecting the contemporary nature of the research topic, particularly in the context of Neurosurgery and Neuropsychology. The database search was completed by January 2025, yielding a total of 198 articles from PubMed, 49 from Scopus, 51 from Web of Science, and 79 from Embase.

**Figure 1.**
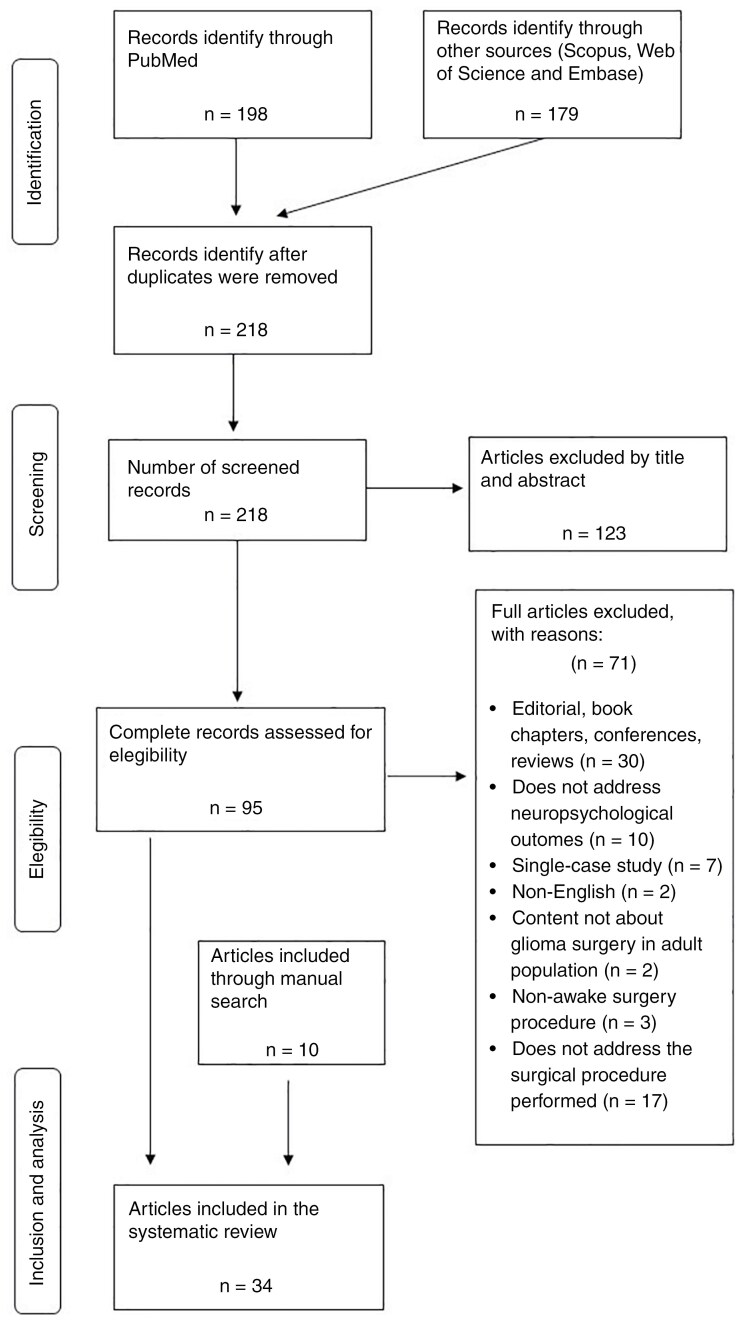
PRISMA (Preferred Reporting Items for Systematic Reviews and Meta-Analyses) flowchart depicting the process of search, screening, inclusion, and exclusion of articles for the current study.

After the first screening, 159 duplicate articles were removed, and a total of 123 articles were excluded based on title and abstract to ensure the selection of relevant studies for further evaluation. Each article was meticulously assessed according to predefined criteria: relevance to the research topic, clarity of reported outcomes, methodological consistency, and the quality of evidence. Specifically, articles that did not focus on awake craniotomy with direct electrical stimulation in adult glioma patients were excluded. Additionally, studies that do not clearly report or assess cognitive recovery outcomes were also excluded. Furthermore, articles that did not align with the methodological framework of the current study, such as those that focus on outcomes unrelated to cognitive and functional recovery, were discarded. The second phase of the screening process involved a thorough review of the 95 full-text articles to identify key variables relevant to our research question, including the number of subjects, their age, the surgical procedure, the participants’ pathologies, the timing of cognitive assessment, the follow-up duration to measure cognitive recovery, and the neuropsychological instruments used. This analysis revealed that several articles did not meet the eligibility criteria, resulting in the exclusion of 71 publications due to the omission of critical data. The comprehensive study selection process is depicted in Figure 1, culminating in the inclusion of 24 articles for the systematic review. Note that in addition to the 24 articles selected from the databases, 10 articles were also included through manual search via references and bibliographies from other articles. This resulted in a total of 34 articles employed for the qualitative analysis conducted in this systematic review. The details of the authors, titles, publication years, and codes are provided in Table 1.

**Table 1.** Articles considered by code, authors, year and title included in the systematic review.

Cod.	Authors	Year	Title
1	Chohan, M.O., Flores, R. A., Wertz, C. and Jung, R. E.^^14^^	2024	“Non-Eloquent” brain regions predict neuropsychological outcome in tumor patients undergoing awake craniotomy
2	Almohamedi, M., Pour-Rashidi, A., Digaleh, H., Zibadi, H. A., Hendi, K., Raminfard, S. et al.^^15^^	2023	Disparity of primary and secondary language outcomes in bilingual patients undergoing resection of glioma of the speech-related regions
3	Donders-Kamphuis, M., Vincent, A., Schouten, J., Smits, M., Docter-Kerkhof, C., Dirven, C. et al.^^16^^	2023	Feasibility of awake brain surgery in glioblastoma patients with severe aphasia: Five case illustrations
4	Gasa-Roqué, A., Rofes, A, Simó, M., Juncadella, M., Rico-Pons, I., Camins, A. et al.^17^	2023	Understanding language and cognition after brain surgery –Tumour grade, fine-grained assessment tools and, most of all, individualized approach
5	Kappen, P.R., van den Brink, J., Jeekel, J., Dirven, C., Klimek, M., Donders-Kamphuis, M. et al.^18^	2023	The effect of musicality on language recovery after awake glioma surgery
6	Lemaitre, A. L., Herbet, G., Ng, S., Moritz-Gasser, S. and Duffau, H.^9^	2022	Cognitive preservation following awake mapping-based neurosurgery for low-grade gliomas: A longitudinal, within-patient design study
7	Reitz, S. C., Beherns, M., Lortz, I., Conradi, N., Rauch, M., Filipski, K. et al.^19^	2022	Neurocognitive outcome and seizure freedom after awake surgery of gliomas
8	Ng, S., Herbet, G., Lemaitre, A. L., Cochereau, J., Moritz-Gasser, S. and Duffau, H.^20^	2021	Neuropsychological assessments before and after awake surgery for incidental low-grade gliomas
9	Noll, K. R., Chen, H., Wefel, J.S., Kumar, V. A., Hou, P., Ferguson, S.D. et al.^21^	2021	Alterations in functional connectomics associated with neurocognitive changes following glioma resection
10	Stålnacke, M., Bergenheim, T. and Sjöberg, R. L.^22^	2021	Neuropsychological function and quality of life after resection of suspected lower-grade glioma in the face primary motor area
11	Bonifazi, S., Passamonti, C., Vecchioni, S., Trignani, R., Martorano, P. P., Durazzi, V. et al.^23^	2020	Cognitive and linguistic outcomes after awake craniotomy in patients with high-grade gliomas
12	Jung, R. E., Wertz, C. J., Ramey, S. J., Mims, R. L., Flores, R. A. and Chohan, M. O.^24^	2020	Subcortical contributions to higher cognitive function in tumor patients undergoing awake craniotomy
13	Motomura, K., Chalise, L., Ohka, F., Aoki, K., Tanahashi, K., Hirano, M. et al.^25^	2020	Neurocognitive and functional outcomes in patients with diffuse frontal lower-grade gliomas undergoing intraoperative awake brain mapping
14	van Kessel, E., Snidjers, T. J., Baumfalk, A. E., Ruis, C., van Baarsen, K., Broekman, M. L. et al.^26^	2020	Neurocognitive changes after awake surgery in glioma patients: a retrospective cohort study
15	Zigotto, L., Annicchiarico, L., Corsini, F., Vitali, L., Falchi, R., Dalpiaz, C. et al.^27^	2020	Effects of supra‑total resection in neurocognitive and oncological outcome of high‑grade gliomas comparing asleep and awake surgery
16	Altieri, R., Raimondo, S., Tiddia, C., Sammarco, D., Cofano, F., Zeppa, P. et al.^28^	2019	Glioma surgery: From preservation of motor skills to conservation of cognitive functions
17	Rijnen, S. J. M., Kaya, G., Gehring, K., Verheul, J. B., Wallis, O. C., Sitskoorn, M. M. et al.^29^	2019	Cognitive functioning in patients with low-grade glioma: effects of hemispheric tumor location and surgical procedure
18	Barzilai, O., Moshe, S. B., Sitt, R., Sela, G., Shofty, B. and Ram, Z.^30^	2018	Improvement in cognitive function after surgery for low-grade glioma
19	Brennun, J., Engelmann, C. M., Thomsen, J. A. and kjøth-Rasmussen, J.^31^	2018	Glioma surgery with intraoperative mapping—balancing the onco-functional choice
20	Motomura, K., Chalise, L., Ohka, F., Aoki, K., Tanahashi, K., Hirano, M. et al.^32^	2018	Supratotal resection of diffuse frontal lower grade gliomas with awake brain mapping, preserving motor, language, and neurocognitive functions
21	Pallud, J. and Dezamis, E.^33^	2017	Functional and oncological outcomes following awake surgical resection using intraoperative cortico-subcortical functional mapping for supratentorial gliomas located in eloquent areas
22	Satoer, D., De Witte, E., Smits, M., Bastiaanse, R., Vincent, A., Mariën, P. and Visch-Brink, E.^34^	2017	Differential effects of awake glioma surgery in (critical) language areas on cognition: 4 case studies
23	Wolf, J., Campos, B., Bruckner, T., Vogt, L., Unterberg, A. and Ahmadi, R.^35^	2016	Evaluation of neuropsychological outcome and “quality of life” after glioma surgery
24	Campanella, F., Fabbro, F., Ius, T., Shallice, T. and Skrap, M.^36^	2015	Acute effects of surgery on emotion and personality of brain tumor patients: surgery impact, histological aspects, and recovery
25	Charras, P., Herbet, G., Deverdun, J., Champfleur, N.,Duffau, H., Bartolomeo, P. and Bonnetblanc, F.^37^	2015	Functional reorganization of the attentional networks in low-grade glioma patients: A longitudinal study
26	Herbet, G., Lafargue, G., Bonnetblanc, F., Moritz-Gasser, S. and Duffau, H.^38^	2013	Is the right frontal cortex really crucial in the mentalizing network? A longitudinal study in patients with a slow-growing lesion
27	Santini, B., Talacchi, A., Squintani, G., Casagrande, F., Capasso, R. and Miceli, G.^39^	2012	Cognitive outcome after awake surgery for tumors in language areas
28	Satoer, D., Vork, J., Visch-Brink, E., Smits, M., Dirven, C. and Vincent, A.^40^	2012	Cognitive functioning early after surgery of gliomas in eloquent areas
29	Papagno, C., Miracapillo, C., Casarotti, A. Romero-Lauro, L. J., Catellano, A., Falini, A. et al.^41^	2011	What is the role of the uncinate fasciculus? Surgical removal and proper name retrieval
30	Sarubbo, S., Latini, F., Panajia, A., Candela, C., Quatrale, R., Milani, P. et al.^42^	2011	Awake surgery in low-grade gliomas harboring eloquent areas: 3-year mean follow-up
**Cod.**	**Authors**	**Year**	**Title**
31	Duffau, H., Gatignol, P., Mandonnet, E., Capelle, L. and Taillandier, L.^43^	2008	Intraoperative subcortical stimulation mapping of language pathways in a consecutive series of 115 patients with Grade II glioma in the left dominant hemisphere
32	Sanai, N., Zahman-Mizradeh, M.D. and Berger, M.S.^44^	2008	Functional outcome after language mapping for glioma resection
33	Bello, L., Galluci, M., Fava, M., Carrabba, G., Giussani, C., Acerbi, F. et al.^45^	2007	Intraoperative subcortical language tract mapping guides surgical removal of gliomas involving speech areas
34	Teixidor, P., Gatignol, P., Leroy, M., Mauset-Aumatell, C., Capelle, L. and Duffau, H.^46^	2007	Assessment of verbal working memory before and after surgery for low-grade glioma

For the analysis of these articles, a two-phase procedure was implemented. In the first phase, the methodological characteristics of the 34 articles were systematically assessed, describing the sample characteristics, histopathology of the patients, lesion location, tumor volume, and the extent of resection (EOR) (Table 2). In the second phase, the information presented in the results sections of each article was synthesized, such as the pre- and post-operative neuropsychological assessment methods used and the follow-up time after awake surgery (Table 3 and Supplementary Table 1).

**Table 2.** Main methodological information of the reviewed articles.

Cod.	Sample size (M/F; n=)	Mean age ± SD	Lesion / Genetic mutation status	Tumor lateralization			Tumor location	Mean tumor volume ± SD (cm^3^)	Neuropsychological functions monitored
				Right hemisphere (n=)	Left hemisphere (n=)				
1	23 / 19	46 ± 18.11	HGG, LGG, cavernous malformations / --	17	25		Frontal lobe (motor cortex), temporal lobe, parietal lobe, and occipital lobe	46.243 ± 57.197	Language, spatial cognition, executive functions, processing speed, memory, verbal fluency, attention, motor skills, social cognition
2	12 / 10	61.6 ± 9.09	HGG, LGG / --	0	22		Frontal lobe, temporal lobe and insular lobe	*------*	Language and speech processing
3	3 / 2	61.4 ± 13.46	HGG, LGG / IDH1	0	5		MFG, SFG, angular gyrus, STG, MTG, and ITG	56.61 ± 14.59	Language and speech processing
4	52 / 35	47.0 ± 15.3	HGG, LGG / --	0	87		Frontal, frontal-insular, frontal-parietal, frontal-temporo-insular, frontal-temporal, insular, parietal, parieto-temporal, temporal, temporo-insular, and temporo-occipital regions	*------*	Language and speech processing
5	28 / 18	36.9 ± 15.03	LGG / --	20	26		Frontal, temporal, parietal, or occipital lobes	39.03 ± 7.53	Language and speech processing
6	85 / 72	38.62 ± 10.55	LGG / IDH	79	78		Frontal lobe, premotor cortex, fronto-insular area, temporo-insular area, temporal lobe, temporo-basal region, insular lobe, insular-frontal area, parietal lobe, and parieto-insular region	114.42 ± 41.85	Executive functions, language and speech processing, social cognition, visuospatial cognition
7	17 / 10	36.1 ± 11.80	HGG, LGG / --	0	27		IFG, anterior-temporal lobe, dSTG, dMtG, and supramarginal gyrus	15.3 ± 6.23	Executive functions, language and speech processing, visuospatial cognition, and social cognition
8	16 / 31	39.2 ± 11.3	HGG, LGG / --	20	27		Frontal, temporal, parietal and insular areas	23.2 ± 23.9	Executive functions, language and speech processing, visuospatial cognition
9	7 / 8	44.6 ± 14.9	HGG, LGG / --	0	15		Frontal, temporal, and parietal lobes	61.1 ± 37.1	Executive functions, language and speech processing
10	1 / 6	51.0 ± 11.62	LGG / --	4	3		Inferior M1	*------*	Executive functions, language and speech processing
11	11 / 8	49.0 ± 10.11	HGG / --	0	19		Precentral, retrocentral, temporal, and insular regions	------	Language and speech processing
Cod.	Sample size (M/F; n=)	Mean age ± SD	Lesion / Genetic mutation status	Tumor lateralization			Tumor location	Mean tumor volume ± SD (cm^3^)	Neuropsychological functions monitored
				Right hemisphere (n=)	Left hemisphere (n=)				
12	35 / 27	49.0 ± 17.5	HGG, metastatic brain tumor, cavernous malformations / --	28	34		Parietal-occipital, parietal, occipital, temporal, and frontal areas	------	Executive functions, language and speech processing, motor skills
13	50	43.7 ± 11.04	LGG / --	16	34		SFG, MFG, IFG, precentral gyrus, and cingulate gyrus	39.9 ± 21.1	Verbal and visual memory, language and speech processing
14	54 / 64	51.69 ± 10.12	HGG, LGG / --	44	118		Frontal, parietal, temporal, and occipital lobes; hippocampus, insula, thalamus, central sulcus, and brainstem	69.10 ± 32.41	Executive functions, language and speech processing, visuospatial cognition, verbal memory
15	18 / 15	53.1 ± 13.2	HGG / --	16	17		Temporal, mesial-temporal, parietal, frontal, temporal-parietal, temporal-insular, frontal, and frontal-parietal	16.24 ± 20.75	Executive functions, language and speech processing, visuospatial cognition, verbal and spatial memory
16	13 / 10	46.0 ± 16.77	HGG, LGG / --	10	13		Frontal-temporal-insular area, frontal tumors near the Rolandic area, frontal-parietal region, frontal-insular area, parietal-insular region, parietal lobe, and temporal lobe	------	Language and speech processing, motor skills, visuospatial cognition
17	46 / 31	50.0 ± 17.32	LGG / --	38	39		Tumor located in or near presumed critical regions for sensorimotor or language functions.	41.6 ± 12.43	Executive functions, language and speech processing, sensory/motor skills
18	28 / 21	35.46 ± 10.57	LGG / IDH1-R132H	23	26		Angular gyri, occipital lobe, supramarginal gyrus, MTG and, frontal lobe	45.69 ± 25.98	Executive functions, language and speech processing
19	43 / 49	36.0 ± 9.83	LGG / --	30	62		Frontal, temporal, parietal, occipital and insular lobes	41.0 ± 21.9	Executive functions, language, memory, attention, mood
20	4 / 5	34.0 ± 8.68	LGG / --	5	4		SFG, MFG, IFG	21.16 ± 13.95	Executive functions, language and speech processing, visuospatial cognition, motor skills
21	59 / 48	40.8 ± 12.5	HGG, LGG / --	27	80		Supratentorial location within or close to eloquent cortical and subcortical regions	65.9 ± 57.6	Executive functions, language and speech processing, visuospatial cognition
Cod.	Sample size (M/F; n=)	Mean age ± SD	Lesion / Genetic mutation status	Tumor lateralization		Tumor location	Mean tumor volume ± SD (cm^3^)	Neuropsychological functions monitored	
				Right hemisphere (n=)	Left hemisphere (n=)				
22	4 / 0	45.5 ± 10.99	HGG, LGG / --	0	4	Cortex parietal, cortex prefrontal, and arcuate fasciculus	54.73 ± 30.75	Executive functions, language and speech processing	
23	12 / 10	55.0 ± 8.66	HGG, LGG / --	0	22	Frontal, parietal, occipital lobes and midline areas	10.3 ± 14.14	Verbal, visual and auditory memory, executive functions, speed processing	
24	36 / 30	49.77 ± 11.77	HGG, LGG, meningioma / --	37	29	Frontal, temporal, and parietal lobes	91.61 ± 61.40	Social cognition, executive functions, language and speech processing, visuospatial cognition	
25	12 / 8	38.9 ± 11.53	LGG / --	20	0	Temporal-insular areas, frontal lobe, temporal-occipital regions, insular lobe, and frontal-temporal-occipital areas	-----	Executive functions, language and speech processing, visuospatial cognition	
26	3 / 7	32.1 ± 9.35	LGG / --	10	0	dmPFC, and fronto-temporal-insular region	96.8 ± 46.8	Executive functions, language and speech processing, visuospatial cognition, social cognition and motor skills	
27	10 / 12	44.91 ± 12.7	HGG, LGG / --	0	22	Parietal, frontal, and temporal lobes	------	Executive functions, language and speech processing, motor skills	
28	19 / 9	41.52 ± 14.39	HGG, LGG / --	0	28	Frontal lobe, temporal lobe, parietal lobe, insular region, frontoparietal region, frontoinsular region, and temporoinsular region	64.54 ± 40.41	Executive functions, language and speech processing, visuospatial cognition	
29	26 / 18	42.18 ± 11.0	HGG, LGG / --	0	44	Uncinate fasciculus, and anterior temporal region,	47.05 ± 1.84	Executive functions, language and speech processing, visuospatial cognition, motor skills	
30	8 / 4	41.9 ± 10.43	LGG / --	3	9	Frontal lobe, fronto-temporo-insular region, temporal lobe, and parietal lobe	------	Language and speech processing	
31	61 / 52	35.0 ± 8.55	LGG / --	0	113	Frontal lobe, temporal lobe, paralimbic region, POTJ, and parietal lobe	------	Language and speech processing	
32	146 / 104	41.2 ± 13.24	HGG, LGG / --	5	245	------	71.0 ± 58.09	Language and speech processing	
Cod.	Sample size (M/F; n=)	Mean age ± SD	Lesion / Genetic mutation status	Tumor lateralization		Tumor location	Mean tumor volume ± SD (cm^3^)	Neuropsychological functions monitored	
				Right hemisphere (n=)	Left hemisphere (n=)				
33	54 / 34	50.0 ± 10.39	HGG, LGG / --	0	88	Frontal lobe, paralimbic region, temporal lobe, and parietal lobe	55.0 ± 59.87	Language and speech processing	
34	12 / 11	34.0 ± 5.20	LGG / --	5	18	SMA, premotor cortex, frontal operculum, operculo-insular region, insular area, parieto-retrocentral area, and parieto-tempro-occipital area	48.2 ± 32.5	Language and speech processing	

dMTG: Dorsal middle temporal gyrus; dSTG: Dorsal-superior temporal gyrus; F: Females; HGG: High grade gliomas; IFG: Inferior frontal gyrus; ITG: Inferior frontal gyrus; LGG: Low grade gliomas; M1: Primary motor cortex; M: Males; MFG: Middle frontal gyrus; MTG: Middle temporal gyrus; POTJ: parieto-occipital-temporal junction; SFG: Superior frontal gyrus; SMA: Supplementary motor area; STG: Superior temporal gyrus.

**Table 3.** Relevant results of the reviewed studies.

Cod.	Pre- and post-operative neuropsychological assessment	Pre-op outcome	Post-op follow-up timing	Post- op outcome	EOR	Adjuvant therapies	Survival rate (%) / Follow- up
1	TOPF, WAIS-II, NAB (Screening, language, spatial, executive), PSI-WAIS-IV, Rey Complex Figure Test, BVMT-R, CVLT-II, COWAT, TMT A/B, Grip Strength, Grooved Pegboard Test, WCST	The patients exhibited an average level of functioning before surgery	24 h and 4-6 months	Immediately after surgery, they showed a decline in cognitive functioning, with notable reductions in attention, language, and executive functioning, while memory and visuospatial skills remained stable. Between 4 to 6 months post-op, there was a notable improvement in all cognitive domains. Cognitive outcomes were worse in patients who underwent radiation therapy.	------	Yes (r + c)	------
2	Validated Persian/Turkish version of Western Aphasia Battery test, Addenbrooke’s Cognitive examination	Language impairment (aphasia)	3 and 6 months	The study found that bilingual patients experienced greater post-surgery language deficits in their secondary language (L2) than in their primary language (L1). While both languages recovered by six months, L2 showed incomplete recovery. Strong preoperative L1 proficiency was key to better outcomes, and L1 consistently outperformed L2 in all assessments.	GTR (n = 16); STR (n = 6)	Yes (r + c)	------
3	Shortened Token Test, BNT, verbal fluency tests, DIMA, DuLIP, ABC, TMT A/B, HVLT, MoCA	Semantic and phonemic paraphasia, executive functions deficits	2 weeks and 3 months	All five patients showed improved language abilities and no further aphasia deterioration at 2 weeks follow-up. Improvements ranged from significant gains, such as one patient achieving near-normal test performance, to moderate progress in others at 3 months post-op.	TR (n = 2); GTR (n = 2); STR (n = 1)	No	------
4	Token Test, verbal fluency test, digit span, BNT, PPTT, semantic pains task	Naming errors, semantic and phonemic paraphasia	24 h and 4 months	Cognitive performance 24 h after surgery significantly worsened for semantic verbal fluency, word-nonword repetition and BNT. 4 months post-op, most patients remained stable or improved.	------	Yes (r + c)	------
5	DIMA, TMT A/B	Prior to surgery, patients displayed a standard level of functioning	3 months and 1 year	General decline in language performance three months after awake glioma surgery. By one year, recovery varied among patients, with some showing improvement while others experienced long-term language decline.	GTR (n = 20)	Yes (r + c)	------
6	TMT A/B, Stroop test, Backward and forward digit span test, DO80, PPTT, reading test, encoding, Rey-Osterrieth Complex Figure, RME, verbal fluency test, R and T, recall test	A significant proportion of patients exhibited cognitive impairments prior to surgery in these cognitive domains: executive functions, language, attention, and verbal episodic memory	3 months	86% of patients showed no cognitive decline, and 10% even experienced improvement. Cognitive functions such as executive functions, psychomotor speed, attention, and verbal episodic memory remained stable in most patients. No patients suffered permanent neurological deficits, and 82.7% returned to their professional activities.	TR (n = 44); STR (n = 80); PR (n = 33)	Yes (r + c)	82.2 % / 3-5 years
7	TAP Alertness, TAP Geteilte (Divided attention), Wortschatztest (Vocabulary), Regensburger Wortflüssigkeitstest (Verbal Fluency), WMS-R, VLMT, BNT, Rey-Osterrieth Complex Figure, Tower of London	Cognitive impairment in at least one domain was observed in 37% of patients, with working memory being the most frequently impaired domain, showing deterioration in 22% of patients.	4-18 months	After surgery, some patients experienced transient aphasia, with the number of impaired patients initially rising but later returning close to baseline after 18 months. A similar trend was observed in executive functioning, where impairments were more prominent in the short term but improved over time, showing significant recovery by the 18-month follow-up.	GTR (n = 15); STR (n = 6); PR (n = 6)	Yes (r + c)	------
8	DO80 task, subtest “code” from WAIS-IV, Stroop task, TMT A/B, verbal fluency test, forward and backwards digit span test, RL-RI16, Rey-Osterrieth Complex Figure, the Taylor Complex Figure, Bells test, line bisection task	Episodic memory was the most affected cognitive domain, followed by slight decreases in phonological and categorical fluency tasks	3 months	At the 3-month follow-up, most cognitive domains showed scores like preoperative levels, except for psychomotor speed and attention, which declined. Many patients experienced stable or improved neurocognitive outcomes, while a small percentage showed slight impairments in at least one cognitive domain.	Supratotal resection (n = 12); TR (n = 16); STR (n = 19)	Yes (r + c)*	100% / --
9	Digit Span, HVLT, TMT A/B, Multilingual Aphasia Examination, fluency test, Token Test, BNT, COWAT	------	3 months	Significant declines in verbal memory, executive functioning, language comprehension, and processing speed. Measures such as HVLT-R for memory, COWA and TMTB for executive functioning, the Token Test for language comprehension, and TMTA for processing speed highlighted these impairments.	GTR (n = 7); TR (n = 3); STR (n = 5)	Yes (r + c)*	------
10	Verbal fluency test, CWIT, BVMT-R, Digit span test	Psychomotor speed and verbal fluency impairments	6 months	Verbal fluency remained stable, with minimal changes observed in category fluency. However, psychomotor speed showed a noticeable decline in tasks involving color naming and reading, indicating impairments in quick cognitive processing.	STR (n = 7)	No	------
11	Laiacona-Capitani Naming Test, BADA, RWL, fluency test, FAB	Naming impairments, verbal memory deficits, lexical retrieval impairments and executive function deficits	3 months and 1 year	At 3 months, naming ability and auditory comprehension showed impairments, with some improvement in naming by 12 months. Verbal memory remained impaired throughout, while word fluency and executive function impairments were significant at 3 months and increased slightly by 12 months.	GTR (n = 6); TR (n = 1); STR (n = 12)	Yes (r + c)	------
12	WASI, NAB, CVLT, Rey Complex Figure Test, TMT A/B, COWAT, Grip Strength, WCST, Processing Speed Index from WAIS-IV	Memory and executive functions deficits	1 week and 4 months	As much subcortical structure volumes, less cognitive impairments after surgery	------	------	------
13	WAIS-IV, WMS-R	Patients did not present significant neuropsychological impairments before surgery.	6 months	Significant improvements were observed in specific cognitive areas, including mean Perceptual Organization scores on the WAIS-III and Visual Memory and Delayed Recall scores on the WMS-R.	Supratotal resection (n = 13); GTR (n = 14); STR (n = 6); PR (n = 17)	Yes (r + c)	------
14	Digit span forward and backwards, TMT A/B, verbal fluency test, Stroop test, 15 WT, Rey-Osterrieth Complex Figure Test, BNT, Token Test	Deficits across multiple domains, including psychomotor speed, visuospatial functioning, language, executive functioning, and memory.	3-6 months	Significant cognitive decline was observed in psychomotor speed and visuospatial functioning, while language, executive functioning, and memory were preserved.	Supratotal resection (n = 12); TR (n = 16); STR (n = 19)	Yes (r + c)*	------
15	DO80 task, verbal fluency test, digit span test, Corsi span test, word list listening, Rey-Osterrieth Complex Figure Test, attentional matrices, line cancelation task, TMT A/B	None of the patients included in the study experienced neurological deficits at conventional neurological examination.	1 week and 4 months	Verbal learning and memory showed significant declines, but both improved to levels above baseline at 4 months follow-up. Constructional praxis was also impaired immediately after surgery.	------	Yes (r + c)	------
16	Comprehension, Expression, Reading, Writing, Pragmatics, Motor speech, Attention, Memory, Problem solving, Visuoperceptive function	------	5 days and 1 month	Early postoperative cognitive impairments were observed in comprehension, expression, reading, attention, memory, and visuoperceptive functions, particularly in patients with left hemisphere tumors. Most functions showed recovery within 30 days, except memory, which remained impaired. Low-grade glioma patients recovered in all domains except memory, while high-grade glioma patients experienced persistent memory impairment.	TR (n = 23)	------	------
17	Verbal memory test, visual memory test, finger tapping test, symbol digit coding test, Stroop test, shifting attention test, continuous performance test, letter fluency test	Notable deficits in cognitive functions such as attention, verbal fluency, and memory	3 months	Significant declines in verbal memory, cognitive flexibility, sustained attention, and processing speed postoperatively. Specifically, they scored lower on verbal memory tests, cognitive flexibility assessments, the Stroop test, and shifting attention tests compared to their preoperative performance. A small percentage of patients demonstrated improvement in their cognitive performance over time.	GTR (n = 4); TR (n = 14); STR (n = 13); PR (n = 3)	Yes (r + c)*	------
18	Digit span backwards and forward, spatial span backward and forward, Rey-Osterrieth Complex Figure Test, Taylor Alternative Figure Test, RAVLT, Hebrew naming test, COWAT, Stroop test	Memory and language deficits	3-28 months	The overall cohort showed significant improvement in cognitive assessments following surgery, particularly in memory and executive functions, while some domains like attention and visuomotor organization did not show significant changes	STR (n = 49)	No	------
19	------	Deficits in executive functions, language, memory and attention	24 h, 6 months, and 12 months	While there was an apparent improvement in mild cognitive deficits after surgery, a significant portion of the cohort continued to experience cognitive deficits, with 31% at 6 months and 20% at 12 months follow-up still reporting cognitive issues.	NTR (n = 51); STR (n = 41)	No	------
20	SLTA, WAIS-III, WMS-R, FAB	Cognitive impairments, particularly in working memory and verbal comprehension	1 month and 6 months	Significant improvements in verbal intelligence, verbal comprehension, generalized memory, and delayed recall at this 6-month follow-up compared to preoperative assessments.	Supratoral resection (n = 9)	No	76%-97% / 3 years
21	BNT, DO80 task, verbal fluency test, PPTT, reading task, Rey-Osterrieth Complex Figure Test, digit span backwards and forward, Bells test, TMT A/B, Stroop test	Some language disturbances, dyscalculia, memory deficits, apraxia, attentional impairments, executive functions deficits	3-5 days and 6 months	Between 3 and 5 days postoperatively, patients experienced challenges such as memory issues affecting recall and learning, attention deficits hindering focus and task completion, executive functioning problems impacting planning and decision-making, and exacerbated language difficulties, including trouble with speaking, understanding, or finding words. However, at the 6-month postoperative follow-up, 89.9% of cognitive impairments improved, 74.4% of patients returned to work, and 30.8% of those with preoperative language disturbances showed significant improvement in language function.	TR (n = 47); STR (n = 33); PR (n = 27)	Yes (r + c)	90%-100% / --
22	BNT, verbal fluency test, AAT, 15 WT, Token Test, digit span test, TMT A/B, Stroop test	2 of 4 patients presented language disturbances, memory problems and executive and attentional functions impairments	3 months, 6 months, and 1 year	Patient 1 experienced a temporary memory deficit at 6 weeks, which resolved by 6 months. Patient 2 showed worsened language and attention/executive deficits at 6 weeks post-surgery. Patient 3, initially impaired in memory and executive functions, exhibited significant recovery by 3 months, maintaining stability at 1 year. Patient 4, despite having a larger tumor, demonstrated intact cognitive performance postoperatively.	STR (n = 4)	Yes (r + c)	------
23	Digit span, VLMT, Figural Memory Test, TMT A/B, WMS-R	Cognitive deficits, but the authors do not specify	Discharge day, and 3 months	Specific deficits in cognitive functions, such as verbal learning and memory, which significantly declined between the preoperative assessment and the day of discharge. These functions showed improvement in the three-month follow-up, reaching levels like preoperative performance.	GTR (n = 22)	Yes (r + c)	------
24	Emotion recognition test, RME, Token Test, FAB, Visual search, stars cancelation, BNT, Rey-Osterrieth Complex Figure, narrative memory, digit span backwards and forward, Corsi spatial span, Italian version of the Toronto Alexithymia Scale	Patients exhibited significant impairments in social cognition tasks such as, theory of mind, alexithymia, and self-maturity	1 week and 4 months	1 week after surgery, patients showed a significant decline in performance, particularly in emotion recognition and theory of mind tasks. However, 4 months after surgery, patients showed significant recovery in emotional recognition and theory of mind abilities, returning to their preoperative levels. Self-maturity scores remained stable across the sessions, indicating no significant change post-surgery.	TR (n = 16); STR (n = 21)	Yes (r + c)	------
25	Verbal fluency test, Stroop test, VOSP, cancelation task, line bisection task	Attentional and visuospatial deficits	2-4 days and 3 months	The patients worsened in lateralized target detection, they made fewer omissions in visuospatial tasks, and no significant results were found in the line bisection task 2 to 4 days after surgery. None of the patients exhibited lateralized deficits in spatial attention tasks three months post-surgery and more than 90% of patients returned to normal socio-professional life without functional deficits.	TR (n = 20)	No	>90% / 6 months-1 year
26	MMSE, digit symbol subtest from WAIS-III, backward and forward digit span, TMT A/B, Stroop test, VOSP, Ideomotor and Reflexive Praxis Protocol, RME	Attention and processing speed impairments	24 h, and 3 months	Immediately after surgery, there was a significant decrease in average mentalizing performance. However, at the three-month follow-up mentalizing scores improved and most patients showed good performance in the other neuropsychological tasks, with many returning to baseline or better than baseline.	Supratotal resection (n = 1); GTR (n = 9)	No	------
27	Raven colored matrix, verbal fluency test, TMT A/B, digit span, Rey-Osterrieth Complex Figure, picture object naming, BADA, limb praxis, orofacial praxis	Language deficits (comprehension and reading difficulties), spatial cognition, verbal memory and word fluency impairments	24 h, and 3-6 months	There was a general decline in cognitive performance, especially in memory and attention immediately after surgery. Six out of 13 patients with preoperative impairments worsened, while four showed improvements. In contrast, only two of nine patients with normal preoperative performance experienced deterioration. Language function, including picture naming, declined immediately after surgery but improved at 3-6 months follow-up, with other language skills showing a similar pattern of initial decline followed by recovery.	------	No	------
28	AAT, BNT, verbal fluency test, 15 WT, TMT A/B, Stroop test, Clock Drawing Test	Language comprehension and production, as well as verbal learning and recall were underscore. Executive functions, including attention, problem-solving, and mental flexibility, were also impaired.	3-4 months	There was a further decline in language and executive functions, particularly in category fluency and letter fluency, as well as tasks like the Trail-Making Test and Stroop tests. However, memory performance improved.	GTR (n = 8); STR (n = 20)	Yes (r + c)	------
29	Raven’s matrices, digit span test, Corsi span test, Word listening learning, Rey-Osterrieth Complex Figure, attentional matrices, TMT A/B, verbal fluency test, sentence comprehension task, orofacial and ideomotor apraxia assessment	Naming, memory, attention, and language deficits and apraxia	24 h, 3 months, and 6 months	Patients who underwent uncinate fasciculus removal experienced persistent naming deficits, with performance on tasks like naming famous faces and objects remaining significantly lower than preoperative levels. Even at follow-up, their scores remained in the impaired range, and reevaluations after 6 months showed no improvement, confirming that the cognitive deficits were long-lasting.	STR (n = 44)	------	------
30	MMSE, Laiacona-Capitani Naming Test, Token Test	Aphasia, motor deficits, amnesia and visual deficits	Discharge day and 3 years	None of the patients showed a worsening in their cognitive functions postoperatively when comparing pre- and post-operative neuropsychological examinations. In fact, some patients demonstrated improvements in specific areas, such as naming abilities and comprehension. The 3-year follow-up demonstrated positive outcomes in terms of neurological stability and improved functional status with no significant cognitive decline reported among the patients.	TR (n = 1); STR (n = 7); PR (n = 4)	No	------
31	BDEA	Only 12 of 115 presented mild language disorders, such as reduction in verbal fluency and slight naming disorders	24 h, 3 months, and 6 months	Immediately after surgery only 2 patients experienced language deficits. However, the rest of the participants, 24 h after surgery maintained or improved preoperative performance. Specifically, at 3 months postoperatively, all patients received full recovery.	TR (n = 37); STR (n = 59); PR (n = 19)	No	------
32	Counting, naming objects, reading single words, repeating complex sentences, writing words and sentences in a paper	Speech arrest, anomia and alexia	1 week, 1 month, 3-6 month and 1 year	All those patients who developed language deficits between 1 and 6 weeks after surgery return to the baseline function or better 3 months after resection. Only 2 out of 5 developed motors hemineglect. In the long-term follow-up, specifically at the 1-year mark, the study indicated that patients who had not shown improvement in their language function were considered to have a permanent language deficit.	GTR (n = 149); STR (101)	------	------
33	Spontaneous speech, verbal fluency test, Famous Face Naming, DO80 task	25 patients (28.4%) exhibited some minor or mild language deficits.	3 days, 1 month and 3 months	While many patients experienced new or worsened language deficits immediately after surgery, a significant proportion showed recovery by the 3-month follow-up	TR (n = 22); STR (n = 6)	------	------
34	DO80 task, BDAE	Verbal working memory deficits	7 days and 3 months	While patients exhibited significant cognitive deficits preoperatively, surgery often resulted in immediate postoperative worsening of these functions. However, with time and rehabilitation, many patients showed recovery and even improvement in their cognitive status three months after surgery.	------	------	------

^*^Patients received the adjuvant therapies after the follow-up period; ------ No details.

AAT, Aachen Aphasia Test; ABC, Aphasia Bedside Check; BADA, Battery for Analysis of Aphasic Deficits; BDEA, Boston Diagnostic Aphasia Examination; BNT, Boston Naming test; BVMT-R, Brief Visual Memory Test Revised; COWAT, Controlled Oral Word Association Test; CVLT, California Verbal learning Test; CWIT, Color-Word Interference Test; DIMA, Diagnostic instrument Mild Aphasia; DO80, Test de Dénomination Orale d’images; DuLIP, Dutch Language Impairment Profile; EOR: Extent of Resection; FAB, Frontal Assessment Battery; GTR, Gross Total Resection; HVLT, Hopkins Verbal Learning Test; MMSE, Mini-Mental State Examination; MoCA, Montreal Cognitive Assessment; NAB, Neuropsychological Assessment Battery; NTR, Near Total Resection; PR, Partial Resection; PPTT, Pyramids and Palm Trees Test; R and T, Rey and Taylor Test; RL-RI16, Rey Auditory Verbal Learning Test—Rey Immediate Recall 16; RME, Reading the Mind in the Eyes; RAVLT, Rey Auditory Verbal Learning Test; RWL, Rey Word List; r + c, radiotherapy and chemotherapy; SLTA, Standard Language Test of Aphasia; STR, Subtotal Resection; TAP, Test Battery for Attention Assessment; TMT A/B, Trail Making Test A and B; TOPF, Test of premorbid functioning; TR, Total Resection; VLMT, Verbal Learning and Memory Test; VOSP, Visual Object and Space Perception Battery; WAIS-IV, Weschler Adult intelligence Scale-IV; WASI-II, Weschler Abbreviated Scale of Intelligence 2^nd^ edition; WCST, Wisconsin Card Sort test; WMS-R, Weschler Memory Scale Revised; 15 WT, 15 Word-Test.

### Inclusion and Exclusion Criteria

The inclusion and exclusion criteria were established based on the PICO (patient population, intervention, comparison, outcome) framework. Inclusion criteria were restricted to articles that reported on adult glioma patients (WHO grade I-IV: patient population) who underwent awake surgery (intervention) and evaluated cognitive and functional recovery during the postoperative period (outcome).

Articles were excluded if the postoperative cognitive and functional outcomes were not clearly reported, not further specified, or were ambiguous. Studies were also excluded if they did not provide original patient data, lacked detailed information on cognitive recovery, or did not report postoperative interventions aimed at improving cognitive function in glioma patients. Additionally, articles were excluded if they were book chapters, comments, abstracts of a conference presentation, single-case studies, editorials, or written in a language other than English. The PRISMA flowchart is presented in Figure 1.

### Participants Characteristics

In Table 2, the main methodological information is synthesized. In the 34 articles selected, the data were collected in order to study the impact of awake surgery on cognitive and functional recovery in adult glioma patients (WHO grades I-IV) during the postoperative period.

The sample size in Table 2 shows the number of subjects, divided by gender, who were included in each of the studies used in the present systematic review. The total number of participants included in this review is 1846 (983 males and 813 females; note that the article coded as 15 did not report the sample size divided by gender). Although most studies had a sample of fewer than 50 participants, four studies include less than 10 patients (3, 10, 20, and 22), and eight publications evaluated 80 to 250 cases (4, 6, 14, 19, 21, 31, 32, and 33). The mean age of the participants ranges from 34.0 to 61.6 (range: 18–81 years).

The lateralization of the tumor was also considered in this study. Right hemisphere lesions were found in 457 participants (24.76%), and left-sided tumors were in 1383 patients (74.92%). The main tumor locations, at cortical or subcortical levels, were specified in all articles selected for the present systematic review, except for 1 of 34 studies (32). In this article, the authors did not report the brain regions where the tumor is located. The primary type of lesion identified was the diffuse low-grade gliomas (LGG, WHO grade II), appearing in 91.18% of the articles (*n* = 31). In addition to LGG, high-grade gliomas (HGG; WHO III and IV) were found in 61.76% (*n* = 21), and other lesions such as cavernous angioma (1), metastatic lesion (12), and meningioma (24) were described (*n* = 1; 2.94%). Among the 34 articles selected for analysis, only three (3, 6, 18) explicitly specify the type of tumor mutation, including detailed information on specific genetic profiles such as IDH mutations (IDH wildtype or IDH mutated) and MGMT promoter methylation status. The mean tumor volume was also reported in 24 out of 34 articles analyzed (70.58%). The mean tumor volumes range from 10.3 to 114.42 cm^3^ (range: 10–130 cm^3^). The rest of the studies (*n* = 10; 29.41%) did not detail the volume of the tumors.

Finally, the neuropsychological functions monitored in each article were identified. All the studies monitored, pre- and post-operatively, language and speech processing. The executive functions were assessed in 21 out of 34 articles (*n* = 61.76%), visuospatial cognition was evaluated in 44.11% (*n* = 15), and sensory/motor skills and praxis were assessed in 8 out of 34 studies (23.53%). Memory abilities and emotional and social cognition were also evaluated, but in a less percentage (*n* = 6; 17.64%/*n* = 3; 8.82%, respectively).

## Results

This section presents the key findings of our systematic review, providing a comprehensive synthesis of the neuropsychological outcomes observed following awake glioma surgery. The results are organized to highlight patterns of cognitive preservation, the influence of surgical techniques, and the temporal dynamics of recovery. By aggregating data from multiple studies, this section aims to elucidate the factors associated with favorable neuropsychological outcomes and identify areas requiring further investigation to optimize patient care.

### Preoperative and Postoperative Neuropsychological Assessment

The studies included in this review consistently utilized preoperative and postoperative neuropsychological assessments to evaluate cognitive and functional outcomes in patients undergoing awake brain surgery. Two of the 34 articles analyzed (16, 19) did not report the neuropsychological tasks performed. The article coded as 16 only describes the cognitive functions assessed and the article coded as 19 does not provide details about the tests employed (Table 3).

Comprehensive information on the neuropsychological tests used in the preoperative and postoperative phases, organized by cognitive domain and by study included in this systematic review, is provided (see Supplementary Table 1 for more details). The main measures of intelligence and overall cognitive functions were the Frontal Assessment Battery (FAB, *n* = 4; 11.76%), the Neuropsychological Assessment Battery (NAB, *n* = 2; 5.88%), and the Weschler Adults Intelligence Scale 4^th^ Edition (WAIS-IV, *n* = 2; 5.88%). The most frequently used tests in the cognitive domain of language and speech were the Verbal Fluency Test (*n* = 16; 47.06%), and the Token Test (*n* = 7; 20.59%). The Boston Naming test (BNT) and the DO80 task were also used but in a less percentage (*n* = 6; 17.64%). The Rey-Osterrieth Complex figure was the most used task for learning and memory (*n* = 12; 35.29%). In addtition to this task, the Weschler Memory Scale Revised (WMS-R) was employed to assess memory skills (*n* = 4; 11.7%). The executive functions were mainly assessed by the Trail Making Test A and B (TMT A/B), the digit span test (backwards and forwards) (*n* = 15; 44.11%), and the Stroop test (*n* = 10; 29.41%). For social cognition, the main test used was The Reading the Mind in the Eyes test (RME) (*n* = 5; 14.7%) and for spatial cognition were the Line Bisection Task (*n* = 2; 5.88%), Bell test (*n* = 2; 5.88%), Corsi span test (*n* = 2; 5.88%), and Raven’s colored matrices (*n* = 2; 5.88%). Finally, the motor skills and praxis were mainly evaluated by the Grip strength task (*n* = 2; 5.88%), the Ideomotor and Reflexive Praxis Protocol (*n* = 2; 5.88%), and the orofacial praxis task (*n* = 2; 5.88%).

### Preoperative Neuropsychological Outcomes

The presurgical cognitive outcomes were described in all the articles of this review, except for those coded as 9 and 23. In the article coded as 9, no details about the preoperative outcomes were labeled. In article 23, the authors reported that the patients suffered from cognitive deficits, but did not specify the type. Standard level of cognitive functioning with an average display in the neuropsychological assessment prior to surgery were described in 4 out of the 34 articles (11.76%) (1, 5, 13, and 15).

Language and speech impairments, such as aphasia, anomia, or speech arrest, were identified in 19 out of the 34 studies selected (55.89%). 14 articles (41.18%) described memory deficits, primarily in episodic and verbal memory. It is important to note that in most of these studies, memory was not the central focus of assessment, and the specific neuropsychological tests used were either not specified or, when listed, the outcomes related to memory were not explicitly reported. Executive function disorders in problem solving or mental flexibility, and attentional deficits were identified in 13 out of the 34 articles analyzed (38.24%). Cognitive deficits related to motor function and praxis, such as psychomotor speed processing disorders or apraxia (*n* = 4; 11.76%), spatial functions, such as navigation problems (*n* = 2; 5.9%), and social cognition and emotional processing (*n* = 1; 2.94%) were the least frequently observed cognitive deficits.

### Postoperative Neuropsychological Outcomes and Comparisons with Baseline

The postsurgical cognitive outcomes were described by all the articles selected for the present systematic review (Table 3). The main cognitive outcomes obtained at two different follow-up timing (immediately after surgery and in a medium/long-term follow-up), will be described and compared with baseline below.

#### Early postsurgical outcomes.

—The early postsurgical outcomes were conducted by 20 out of the 34 studies reviewed (58.82%). Seven articles (1, 4, 19, 26, 27, 29, 3) performed the neuropsychological assessment session 24 h after surgery; 10 studies (3, 12,15, 16, 21, 24, 25, 32, 33, 34) conducted the evaluation session between 2 days and 1-week post-op; and 2 articles (23, 30) the discharge day.

Fifteen out of the 20 articles (1, 4, 15, 16, 20, 23, 24, 25, 26, 27, 29, 31, 32, 33, 34) that conducted early postsurgical evaluations (75%), describe slight declines in cognitive functions after surgery in comparison with the presurgical performance. Note that article coded as 4 reported that patients’ cognitive performance in semantic verbal fluency get worse in the immediately post-op assessment. Similarly occurred in article coded as 29. Patients who underwent uncinate fasciculus removal showed a significant decline in naming tasks, with performance remaining markedly lower than preoperative levels. Only one article (1) indicated that memory and visuospatial skills remained stable after surgery. All patients exhibited scores similar to those obtained during baseline. Although most studies reported deteriorations immediately after surgery, the articles coded as 3, 19, and 30 informed improvements in all cognitive domains at the post-acute neuropsychological assessment.

#### Mid- and *l*ong-*t*erm *f*ollow-up.

The ongoing evaluation was reported in 33 out of the 34 articles selected for this review (97.6%). This ongoing neuropsychological evaluation was mainly performed between 3 months and 6 months post-op (*n* = 27; 81.82%). Five articles (15.16%) (1, 4, 7, 12, 24) conducted the cognitive evaluation at 4 months post-op, and 4 studies (12.12%) (16, 20, 32, 33) informed a follow-up on cognitive abilities 1 month after surgery.

The long-term follow-up was described in 23.53% (*n* = 8) of articles. Most articles that report long-term assessments focus on the one-year timeframe (*n* = 5; 62.5%). Nevertheless, 3 articles (7, 18, 30) reported a follow-up on cognitive functions extending beyond one year. The article coded as 7 informed about 18-month follow-up. In the article numbered as 18, the long-term assessment was conducted at 28-month follow-up, and similarly, the article coded as 30 performed the long-term neuropsychological evaluation at 3 years follow-up.

Regarding the postoperative outcomes during these evaluation periods, 22 out of 34 articles (64.7%) reviewed demonstrated cognitive stability or improvements during the medium- and long-term evaluations when compared to baseline measures. It is noteworthy that in 5 articles (3, 6, 25, 31, 33), complete recovery was observed from both preoperative deficits and those identified in the neuropsychological assessments conducted immediately after surgery. Furthermore, two articles (6, 21) emphasize that most patients restarted their professional activities following the cognitive outcomes observed in the long-term evaluation. Despite these favorable outcomes, 9 articles (1, 2, 5, 8, 10, 14, 16, 19, 32) indicate that, although long-term improvements have been observed, certain cognitive domains continue to exhibit deficits or deteriorate, as observed in the article coded as 1, where patients who underwent adjuvant therapies showed progressively lower scores in neuropsychological assessments. Furthermore, it is remarkable that 2 articles (5, 32) reported the cognitive deficits that persist during the medium- and long-term follow-up period as permanent impairments.

#### Surgical *r*esults.

The type of resection, extent of resection, adjuvant therapies, and survival rates are detailed in Table 3.

The EOR was described in 28 out of the 34 studies analyzed (82.35%). 21 out of the 28 performed a subtotal resection (STR); 14 articles (50%) conducted a total resection (TR); 12 (42.86%) carried out a gross total resection (GTR); 7 studies (25%) developed partial resections; and 5 articles (17.85%) conducted a supratotal resection. The remaining 6 out of the 28 articles (1, 4, 12, 15, 27, 34) did not provide information concerning the EOR.

Regarding the adjuvant therapies (radiotherapy and chemotherapy), 18 articles (52.9%) reported that patients underwent these treatments. It is important to note that, in four studies (8, 9, 14, 17), patients were subjected to these treatments following the neuropsychological assessment during the follow-up, due to the cognitive decline that adjuvant therapies may induce. Eight studies (3, 10, 18, 19, 20, 25, 26, 27, 30, 31) refused to use the adjuvant therapies, and 5 (12, 16, 32, 33, 34) did not report details about complementary therapies.

Finally, the survival rate was only provided by 5 out of the 34 articles (6, 8, 20, 21, 25). Two of them (6, 20) reported a survival rate between 70% and 85%, referring to the proportion of patients alive during the follow-up period of 3 to 5 years in which the survival rate was assessed in these studies. Three of them reported survival rates of 90% (21, 25) and even up to 100% (8) with survival outcomes evaluated over follow-up periods ranging from 6 months to 1 year.

## Discussion

This systematic review examines the neuropsychological outcomes of awake craniotomy for glioma resection, emphasizing the importance of preserving cognitive and social cognitive functions. Over the past two decades, extensive research has explored the impact of this surgical approach on cognitive recovery, revealing the need for comprehensive pre- and postoperative neuropsychological assessments. The review highlights the variability in cognitive and social cognitive outcomes, with many patients demonstrating recovery within 3 to 6 months post-surgery, though long-term follow-ups suggest continued neuroplastic adaptation. By synthesizing findings from multiple studies, this review provides insights into the functional anatomy of critical brain networks, guiding neurosurgeons and neuropsychologists in optimizing surgical planning and rehabilitation.

Recent studies^9,10,20^ have demonstrated that awake craniotomy significantly influences cognitive and emotional recovery, emphasizing the need for comprehensive assessments and tailored rehabilitation strategies that address the brain’s nuanced responses to surgical intervention. In this context, a systematic review^47^ demonstrates that intraoperative mapping of the right hemisphere can identify cognitive and social cognitive functions at risk during surgery, highlighting the role of neuropsychological tasks in minimizing postoperative deficits. Social cognitive functions may also be assessed in the left hemisphere, as their lateralization can vary depending on the task and individual differences, and neuroplasticity may support interhemispheric compensation following resection.^11^ Together, these findings emphasize the importance of considering both hemispheres during surgical planning and neuropsychological evaluation, recognizing the dynamic and adaptable nature of social cognitive networks. The variability in cognitive and emotional outcomes following awake craniotomy is evident across the reviewed studies. While some patients experience significant cognitive improvement within the first few months post-surgery,^14,17^ others exhibit persistent deficits.^27,35^ In addition to cognitive domains, social cognition also shows a range of outcomes post-surgery. The review highlights that improvements in social cognitive functions, such as emotional processing and empathy, are often noted alongside enhancements in executive control and attention.^10,20^ However, similar to cognitive improvements, the changes in social cognition can be inconsistent, with some patients struggling to regain their social cognitive skills postoperatively, reflecting the complexities of recovery as influenced by factors like the extent of resection and individual differences in brain plasticity.^9,11^ This inconsistency underscores the importance of closely monitoring cognitive and emotional performance both immediately and overtime. The timing of neuropsychological assessments is crucial for understanding cognitive and emotional recovery, as a study^20^ revealed that the results of rehabilitation evaluations are heavily influenced by the timing of their administration. Herbet et al. (2013)^38^ and Lemaitre et al. (2022)^9^ further emphasize that cognitive and emotional recovery may extend beyond the commonly used 3- and 6-month postoperative periods, supporting the notion that neuroplastic adaptations continue for several months. Extending follow-ups to 1 year post-surgery could provide a more comprehensive understanding of the cognitive and emotional progression and long-term patient outcomes.

A key framework for interpreting cognitive and social cognitive recovery after glioma resection is the meta-networking model, which posits those cognitive functions, such as motor skills, language, executive function, and social cognition, are dynamically interconnected rather than isolated.^3^ Damage to one area may lead to compensatory adaptations or disruptions in others due to mechanisms like diaschisis, where an injury affects functionally connected but otherwise intact regions. This model underscores the brain’s remarkable capacity for neuroplasticity, allowing preserved networks to take over impaired functions and promote recovery. Studies included in this review^23,25^ demonstrate that effective intraoperative monitoring and mapping optimize tumor resection while minimizing cognitive and emotional impairment. Resection of gliomas near functionally critical network hubs^15,30^ highlights that postoperative cognitive outcomes are influenced not only by the extent of resection but also by how well neural networks are able to reorganize. For instance, when language-related structures are compromised, executive function networks may adapt to support communication, showcasing the brain’s inherent adaptability.^23,25^

Functional reorganization is pivotal in postoperative cognitive and social cognitive recovery, and neuropsychological assessments are essential in tracking this process. They provide critical insights into a patient’s preoperative cognitive profile, identify at-risk functions, and monitor recovery over time. Two studies^9,10^ emphasize that assessing multiple domains, including language, executive function, memory, social cognition, and attention, is essential for developing individualized rehabilitation plans and understanding the trajectory of cognitive and emotional recovery after awake brain surgery. These tailored interventions leverage intact cognitive and social cognitive functions to compensate for deficits, ensuring a more effective recovery progression and improving overall quality of life.^11^

This review highlights the importance of molecular factors like IDH mutation status, which can influence long-term neurocognitive recovery after glioma surgery. IDH mutations, linked to better prognosis and unique biological traits, may affect neural plasticity and rehabilitation.^48^ Considering IDH status supports personalized assessment and intervention, improving prognostic accuracy and enabling tailored rehabilitation to optimize patient outcomes. The extent of resection (EOR), adjuvant therapies, and survival rates are key factors influencing long-term patient outcomes. A strong correlation has been found between higher EOR and improved survival rates, reinforcing the importance of balancing maximal tumor removal with the preservation of neurological function, a principle central to awake surgery, which enables real-time cognitive monitoring during resection.^3,9–11,39^

Beyond surgical factors, adjuvant therapies such as radiotherapy and chemotherapy play a complex role in cognitive recovery. While they contribute to prolonged survival, they may also induce cognitive decline, particularly when administered shortly after surgery.^9,20–22,25,35,36^ Despite these challenges, the reported survival rates in the reviewed studies, exceeding 90% in a significant proportion of patients, highlighting the impact of advancements in surgical techniques and the benefits of multidisciplinary care strategies.^37,41^ Integrating surgical, oncological, and neuropsychological care could further optimize long-term patient outcomes and quality of life.

In this context, a deeper understanding of cognitive and emotional recovery through the meta-networking model provides a foundation for refining rehabilitation strategies that harness neuroplasticity. Research suggests that enhancing specific cognitive functions, such as attention, could facilitate recovery in interconnected domains like language.^6,7^ Future studies should focus on developing rehabilitation protocols that leverage preserved cognitive functions to compensate for deficits, ultimately improving post-surgical quality of life for glioma patients. Given the variability in patterns of cognitive and emotional processing progression, standardized postoperative follow-up protocols are essential. Extending follow-up periods, as emphasized in multiple studies,^9,20,38^ could offer a clearer understanding of long-term recovery patterns. Standardizing the timing of neuropsychological assessments would not only reduce discrepancies in reported outcomes but also facilitate cross-study comparisons, ensuring more accurate and useful insights into cognitive rehabilitation, including social cognition.

### Strengths and Limitations

This systematic review presents several strengths that contribute to its scientific relevance and clinical applicability. First, it synthesizes data from 34 studies, offering a comprehensive analysis of neuropsychological outcomes following awake surgery for glioma patients. The inclusion of studies with diverse methodological approaches allows for a broad understanding of cognitive recovery trajectories, reinforcing the importance of standardized yet individualized neuropsychological assessments. Additionally, by considering multiple cognitive domains, including executive functions, memory, visuospatial processing, and social cognition, this review provides a more holistic perspective on postoperative cognitive recovery beyond language preservation. The discussion of follow-up timing and the impact of surgical and oncological factors further enhances the study’s clinical significance, offering valuable insights for optimizing long-term patient care.

Despite these strengths, certain limitations must be acknowledged. The variability in neuropsychological assessment tools and follow-up durations across studies introduces methodological inconsistencies, making direct comparisons challenging. Many studies lack long-term cognitive assessments beyond 6 months, which limits the understanding of prolonged neuroplastic adaptations. Additionally, differences in tumor location, histological subtypes, and extent of resection introduce heterogeneity that may influence cognitive outcomes. Another limitation is the potential underreporting of cognitive deficits in studies relying on less sensitive neuropsychological measures, which may also overlook challenges related to social cognition. Furthermore, while the review considers the effects of adjuvant therapies, their specific impact on cognitive recovery remains difficult to isolate due to variations in treatment protocols. Finally, given that this review exclusively included studies involving intraoperative direct electrical stimulation mapping during awake surgery, the lack of comparison with non-mapped control groups limits the ability to isolate the specific contribution of mapping to cognitive outcomes. Inclusion of non-mapped cohorts would have introduced significant heterogeneity, but this remains an important gap in the literature that future controlled studies should address.

## Future Directions

This review highlights the need for a more unified and theoretically grounded approach to neuropsychological assessment in glioma patients undergoing awake surgery. The wide variability in current protocols regarding structure, timing, and content not only limits comparability across studies but also hinders the accumulation of broadly applicable knowledge.

To overcome these limitations, future efforts should focus on methodological harmonization, while maintaining the flexibility required to adapt to individual patient profiles and clinical contexts. Central to this goal is the development of assessment methods that are both clinically meaningful and responsive to the dynamic nature of brain function throughout the perioperative period. Achieving this balance involves reconciling scientific rigor with the practical constraints of intraoperative and perioperative environments.

In this context, intraoperative tasks should follow key methodological principles: be brief and efficient to fit surgical time constraints, include equivalent items to minimize learning effects, and avoid binary response formats like yes/no that risk chance-level responses. They must reliably elicit observable behaviors such as naming, repetition, or gestures and be sensitive enough to detect subtle, real-time cognitive fluctuations during tumor resection.^49^ Such design considerations are essential to developing assessment tools that are not only clinically meaningful but also adaptable to the dynamic and constrained nature of the perioperative environment.

Although these features are crucial intraoperatively, the same methodological principles should also inform the design of postoperative assessments. Therefore, building on this intraoperative framework, a functionally tailored, network-based approach should be extended across the entire perioperative continuum, ensuring consistent and personalized neuropsychological care throughout the patient’s surgical trajectory.^11^ By integrating psychometrically robust tests that are valid, reliable, and offer multiple forms or well-documented repeatability, while remaining aligned with the neural networks at risk, clinicians can ensure continuity of care that not only informs surgical decisions but also supports long-term functional outcomes through targeted rehabilitation.

An effective way to strengthen the ecological validity and functional relevance of neuropsychological assessments across the entire perioperative period is to consistently employ tests that have demonstrated sensitivity not only to the cortical and subcortical regions stimulated during awake surgery but also to specific cognitive domains. This consistency facilitates more accurate detection and monitoring of clinically meaningful cognitive changes over time. Building on the need for sensitive and clinically relevant assessments, some of the following tests that have proven effective during intraoperative mapping could also be valuable for preoperative and postoperative evaluations. These include picture naming tasks (DO-80 task, Boston Naming Test) for lexical retrieval; scene description tasks to assess spontaneous speech; Digit Span and Letter–Number Sequencing for verbal working memory; and the Pyramids and Palm Trees Test (PPTT) for semantic cognition. Additionally, tasks targeting syntax, reading, inhibition (Stroop test), social cognition (RME), praxis, and visuospatial skills (line bisection task) further align assessments with the functional anatomy and cognitive domains involved.

To ensure these tasks can be effectively integrated into clinical practice, their use must be adapted to each patient’s condition and context. Employing a tiered assessment strategy that begins with brief screenings and expands only when necessary helps balance diagnostic accuracy with patient comfort. Future protocols should focus on flexible, validated formats that can be used across a variety of clinical settings. Reaching clinical consensus on these approaches will be essential for establishing a standardized yet adaptable model of neuropsychological care.

## Conclusions

This systematic review highlights the crucial role of long-term neuropsychological assessment in glioma patients undergoing awake brain surgery. Unlike previous studies that have primarily focused on short-term cognitive and emotional outcomes. Our review highlights the variability in long-term patterns of cognitive and social cognitive progression and the need for standardized follow-up protocols. While many patients experience cognitive recovery within the first 3 to 6 months post-surgery, some exhibit persistent deficits in executive function, language, or theory of mind. These findings emphasize that cognitive and social cognition recovery is a dynamic and individualized process that extends beyond the immediate postoperative period, reinforcing the importance of prolonged neuropsychological monitoring.

The observed heterogeneity in recovery patterns further reinforces the importance of developing standardized follow-up protocols capable of capturing the complex and evolving nature of neuropsychological outcomes in this patient population. While a substantial number of patients demonstrate cognitive improvement within the first 3 to 6 months post-surgery, others continue to experience lasting impairments in areas such as executive function, language, or theory of mind. These findings suggest that cognitive and social cognitive recovery is not a uniform process but rather a dynamic and individualized trajectory, extending well beyond the immediate postoperative period. Together, these insights emphasize the necessity of prolonged and personalized neuropsychological monitoring, as well as the integration of emerging cognitive domains into future research frameworks, to enhance the accuracy, interpretability, and clinical utility of long-term outcome assessments.

The variability in cognitive and emotional outcomes following awake brain surgery can be better understood through the meta-networking model, which suggests that cognitive and emotional functions do not rely on isolated brain regions but rather emerge from interactions within large-scale neural networks. When a specific area is damaged, functionally connected regions may reorganize to compensate, leading to diverse recovery patterns. This perspective underscores the importance of intraoperative mapping beyond language preservation, advocating for assessments of executive function, memory, or social cognition to ensure a more comprehensive approach to cognitive and emotional preservation during surgery.

Building on this framework, long-term evaluation is essential not only to track recovery but also to guide rehabilitation strategies that harness neuroplasticity. To optimize patient outcomes, follow-up protocols must combine standardization with flexibility, adapting to each patient’s clinical profile. Furthermore, the limited reporting of molecular characteristics, such as IDH mutation status and MGMT promoter methylation, across existing studies highlights the need for more comprehensive genetic reporting to enhance the interpretability and clinical relevance of cognitive outcome data.

Future research should aim to validate flexible, structured neuropsychological assessment protocols. Our proposed model provides a modular, adaptable framework based on clinical variables and patient needs. Tiered testing strategies and accessible formats, including remote and bedside options, can minimize patient burden and support a standardized, patient-centered approach to long-term follow-up in glioma care.

## Supplementary material

Supplementary material is available online at *Neuro-Oncology Practice* (https://academic.oup.com/nop/).

npaf075_Supplementary_Table_1
